# Retroperitoneal Hematoma as a Complication of Central Venous Catheterization: A Case Report

**DOI:** 10.1002/ccr3.72591

**Published:** 2026-04-21

**Authors:** Sadegh Abaei Hasani, Samin Shokravi, Payam Fattahi, Niki Tadayon

**Affiliations:** ^1^ Department of General Surgery, School of Medicine Shahid Beheshti University of Medical Sciences Tehran Iran; ^2^ Research Associate, Department of Surgery University of Miami, Miller School of Medicine Miami Florida USA; ^3^ School of Medicine Shahid Beheshti University of Medical Sciences Tehran Iran

**Keywords:** case report, central venous catheterization, iatrogenic vascular trauma, retroperitoneal hemorrhage

## Abstract

Central venous catheter (CVC) insertion is generally straightforward but carries risks. While most complications are minor, rare serious events can occur, such as retroperitoneal hematoma. This report describes a patient who developed this complication after femoral CVC insertion and outlines the diagnostic approach, management, and important clinical insights.

## 1 Introduction

Central venous catheter (CVC) insertion is a common procedure with known complications. Retroperitoneal hematoma, however, is a rare but potentially life‐threatening event. This case report discusses a unique instance of retroperitoneal bleeding following femoral vein catheterization in a patient without coagulopathy, highlighting diagnostic and management challenges.

We can categorize its main applications into three domains: first, for monitoring central vein pressure; second, for fluid administration. Lastly, when we fail to apply for any other venous access, despite its many applications, there are some complications, according to several case reports, which enlighten the importance of indicated application and observation for further adverse effects [1].

One of the most important life‐threatening conditions in coagulopathy patients is the major bleeding complications secondary to CV line application [2, 3]. In 2006, Ahmad L Almori reported a rare case of bleeding in the area behind the abdominal cavity caused by a misplaced CVC in a patient who had a history of blood‐clotting issues. In this paper, we report a rare case of retroperitoneal hemorrhage secondary to a misplaced central venous catheterization in a patient without any coagulopathy history.

## 2 Case History

### 2.1 Clinical Presentation

A 65‐year‐old woman with a recent history of direct trauma toward the neck of the femur went through ORIF (open reduction and internal fixation) for intertrochanteric fixation 2 days ago in an orthopedic surgery center. The challenge facing the clinicians in the management of this patient was the absence of proper peripheral venous access due to recent intensive chemotherapy; therefore, a left femoral vein CVC was inserted. The patient has received isotonic saline and pack cells through the mentioned catheter. Following surgery, despite the administration of whole blood pack cells, the patient experienced a drop in hemoglobin levels and worsening abdominal pain. The potential for a retroperitoneal hematoma was noted, and it was the reason she was referred to our vascular surgery center.

Upon admission to our hospital, the patient appeared slightly ill, was conscious but was capable of responding to our questions, and had pale conjunctiva. Vital signs were a systolic blood pressure of 120 mmHg, a pulse rate of 92, an SpO2 of 97%, and a respiratory rate of 17. The puncture site for trying to take vein was visible on the left and right sides of the neck. The chest wall had normal expansion and no reduction in lung sounds. The abdomen was soft but had tenderness in the left lower quadrant (LLQ). All four limbs were warm, and their distal pulses were symmetric. The CVC entrance site was on the left inguinal, and there was no hematoma or ecchymosis around it.

The patient's hemoglobin levels had dropped to 7 g/dL 1 day after orthopedic surgery, so one whole blood pack cell was injected through the same CV line at Akhtar Hospital. However, the patient's hemoglobin level did not improve but rather decreased from 7 g/dL to 6 g/dL. Another pack cell was injected in the same way, but there was no significant improvement, and abdominal pain gradually began to appear after the pack cell injection.

### 2.2 Differential Diagnosis/Investigations and Treatment

**FIGURE 1:**
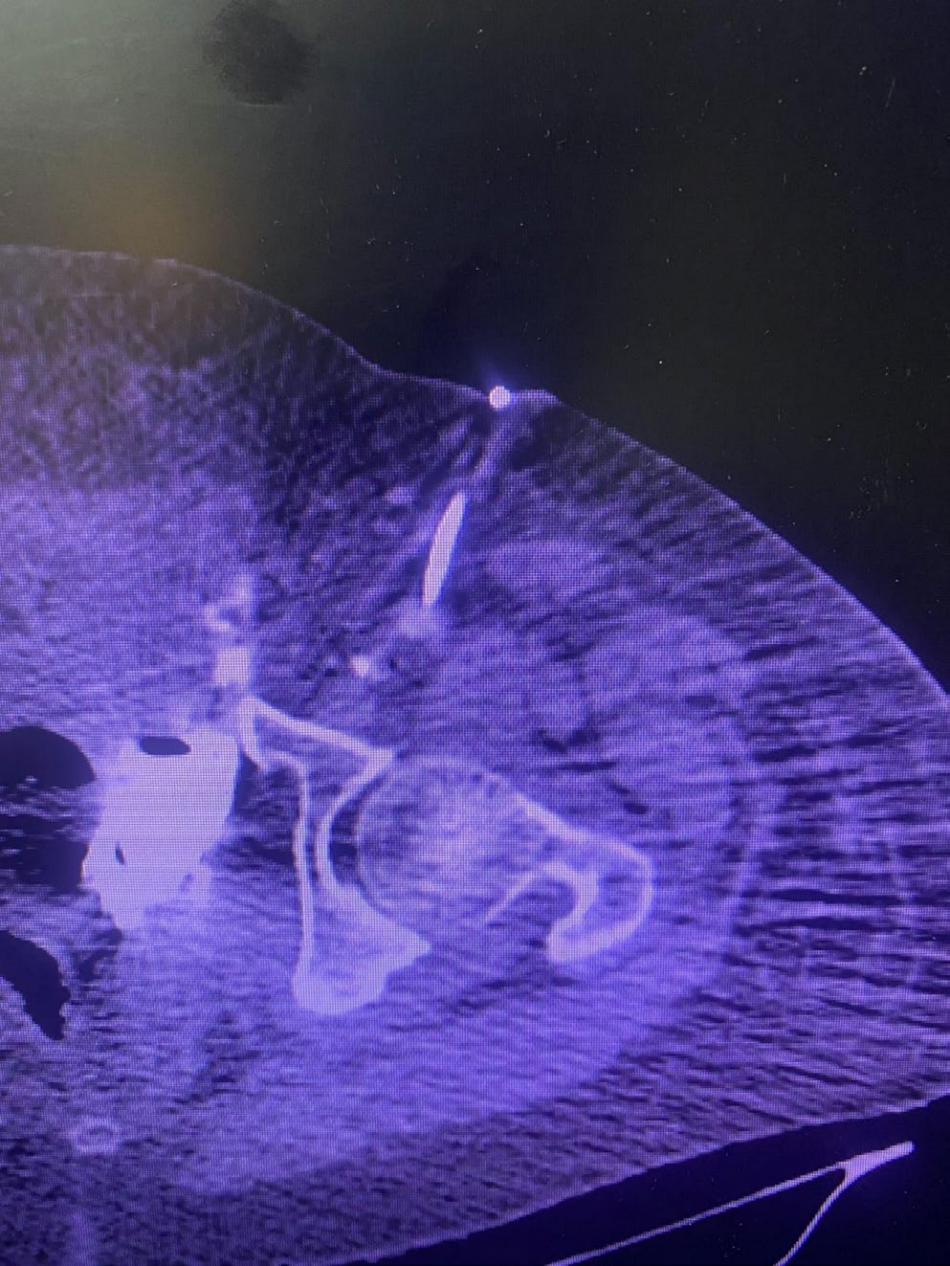
A CT angiography revealed a misplaced central vein catheter in the retroperitoneal space.

**FIGURE 2:**
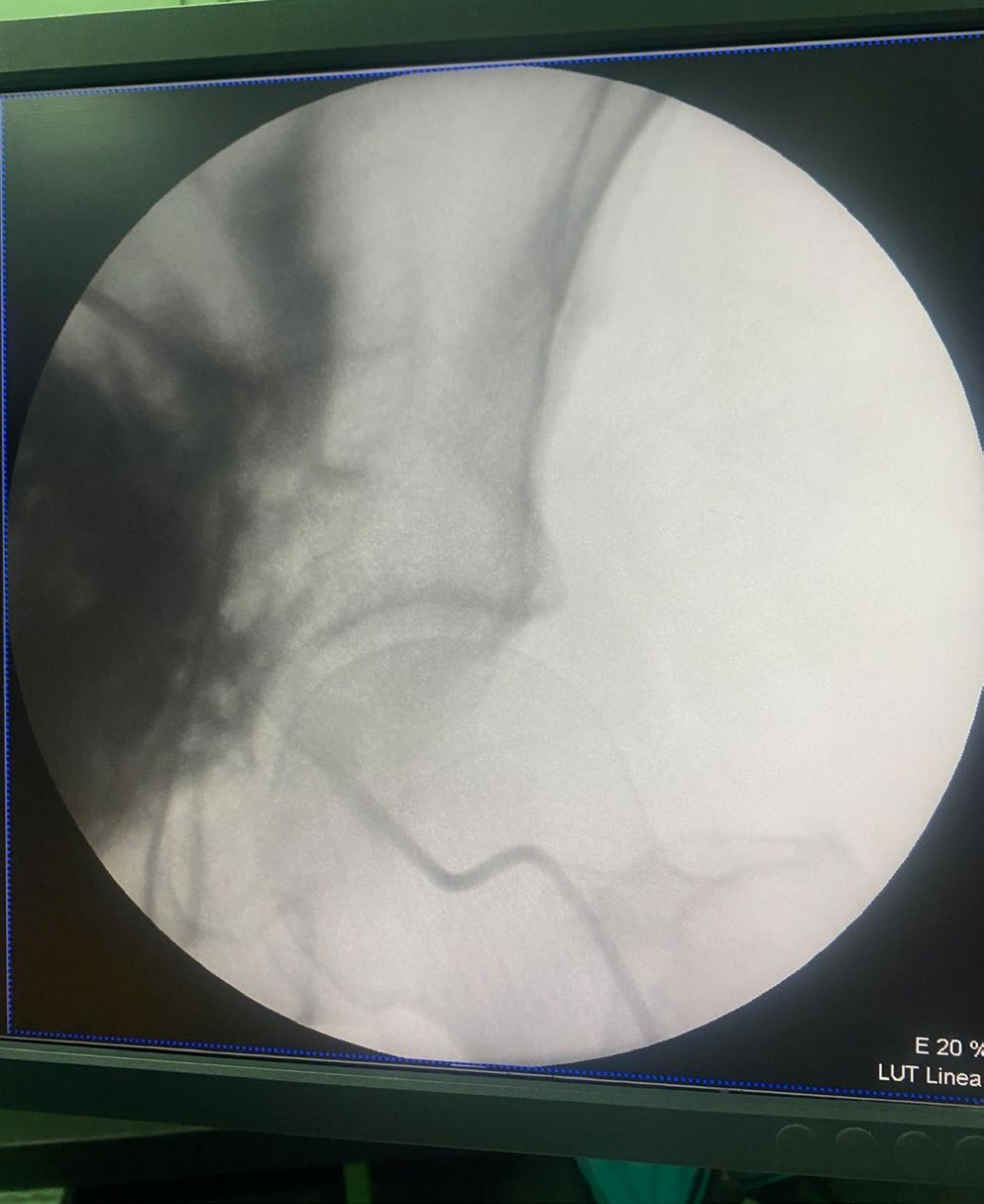
Angiography demonstrated that contrast blushed into the retroperitoneal space.

We tapped the CV line to examine its position, but no blood returned. A slight repositioning resulted in venous blood return, so the left jugular vein was provided for venous access. The patient's condition necessitated a CT scan, so 20 mL of contrast was injected through the femoral CV line, and CT angiography was performed (Figure 1), but due to the patient's worsening abdominal pain, the procedure was discontinued. The CT angiography revealed that the CV line penetrates and passes through the common femoral vein. Contrast has spread in all of the vessels and leaked in the retroperitoneal space (Figure 2). The CT scan result has proven the retroperitoneal hematoma. Tenderness and rebound tenderness of the left lower abdominal quadrant were reported. The physical exam and imaging results led to the patient's admission to the operating room. The patient provided written informed consent for the surgical intervention and research purposes, after which she proceeded to the operating room.

In a hybrid operating room, the patient received local sedative anesthesia for angiography. Ten milliliters of iohexol, diluted to 50%, was injected through the central line of the CVC. Because of the artifact caused by the CT contrast, angiography did not yield sufficient data. The only useful information obtained from angiography was the presence of contrast flush. We assumed the catheter's side hole would be in the artery and its tip in the retroperitoneal region. The catheter was gently retracted while being repeatedly tapped. The CV line artery injury was confirmed after 2 cm of holding the line back, and arterial blood returned.

**FIGURE 3:**
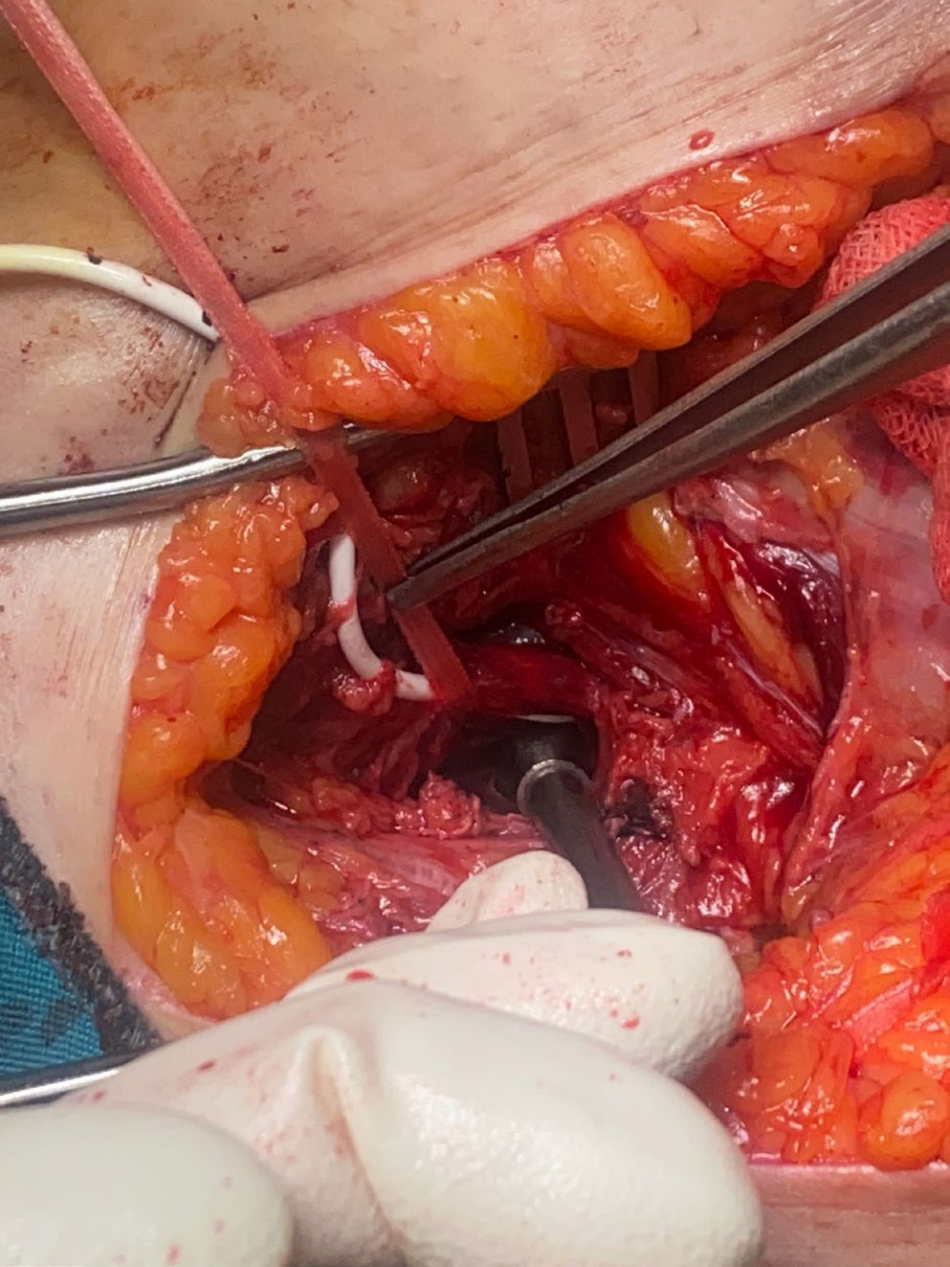
Misplaced central venous catheter.

Given the patient's tenderness and rebound tenderness, and considering the need for comprehensive data regarding any potential vascular injuries above the inguinal ligaments, we took the proactive step of exploring the retroperitoneal space alongside the inguinal exploration. A hockey stick incision on the left side opened up exciting opportunities for us to explore the retroperitoneal space. Epidermis, dermis, hypodermis, and scarpa fascia were incised. The internal and external oblique muscles' fascia was split, and 500 cc of clot was extracted from the retroperitoneal space. Iliac artery control was performed, the iliac artery and vein were intact, and there was no evidence of active hemorrhage in the retroperitoneal space. The hockey stick incision was extended in order to explore the femoral vein and artery. Vascular tape was used to explore and control the common femoral artery (CFA); the CV line passed through the CFA without any interruption (Figure 3). The entry site was in the anterior of the CFA, inferior to the inguinal ligament, and the exit site was on the posterior side and proximal. The totally occluded clamp was stuck on the common iliac artery, and another clamp was stuck distal to the CFA.

The CV line was extracted, and the femoral artery was repaired with prolene 6‐0 fiber. After repairing, distal limb pulses were normal. A hemo‐bag drainage was placed in the retroperitoneal space and the inguinal space. Muscles were repaired with 1 vicryl fiber. The derm was repaired with 3‐0 vicryl fiber. The skin was repaired with 3‐0 plastic fiber. The patient was extubated and, with a generally good condition, was referred to the ICU.

## 3 Conclusion and Results (Outcome and Follow‐Up)

On the initial postoperative day, the patient's overall condition was satisfactory, the most recent hemoglobin level was 8.1, and the left lower extremity pulse was unremarkable. No bleeding or hematoma was observed at the surgical site. An orthopedic consultation was conducted to further treatment. A CT scan conducted 1 week post‐surgery revealed no indications of retroperitoneal hematoma.

In conclusion, life‐threatening retroperitoneal hemorrhage associated with femoral vein catheterization can occur. To avoid puncture of incompressible retroperitoneal vessels during femoral vein cannulation, the needle should be inserted into the femoral triangle in such a fashion that it penetrates the vein caudal to the inguinal ligament; the needle should not be inserted at or immediately below the inguinal ligament and should not be advanced too far at a low angle. The seriousness of our case suggests the need to consider prompt surgical repair whenever a large‐bore catheter or needle is placed in an incompressible major artery.

## 4 Discussion

In this paper, we report a rare case of retroperitoneal hemorrhage secondary to a misplaced central venous catheterization in a patient without any coagulopathy history. CVCs are commonly used in critically ill patients and those undergoing major surgery [4, 5]. Unfortunately, various adverse events can result from their use, which is associated with increased morbidity, mortality, and healthcare costs [6, 7]. Mechanical complications are reported in 5%–19% [1, 4, 8], infectious complications in 5%–26% [4, 7, 9], and thrombotic complications in 2%–26% of patients undergoing CVC placement [4].

Factors associated with a decreased risk of mechanical complications include (1) increased operator experience; (2) site of insertion rates of mechanical complications with femoral catheterization are higher than subclavian or internal jugular sites; (3) successful recognition of risk factors for difficult catheterization (e.g., unfavorable patient's body habitus, prior failed catheterization attempts, need for catheterization at sites of prior surgery, skeletal deformities, and scaring); (4) ultrasound guidance; (5) fewer insertion attempts; and (6) avoidance of scheduled, routine replacement of catheters at new sites [4].

Various recommendations have been made as to the site for insertion of the needle and the angle for advancement of the needle. Regarding the site for insertion, several authors have recommended a site approximately 2 to 3 cm, or two finger widths, below the inguinal ligament [10, 11, 12, 13], whereas others have suggested insertion of the needle at sites much closer to the inguinal ligament, that is, at the inguinal skin crease, approximately 1 cm below the inguinal ligament or at, or just below, the inguinal ligament [14]. Most of these authors have recommended advancing the needle at angles greater than 45 degrees from the frontal plane, including 45 , 45–60 , 45–70 , and 90 , whereas only a single previous reference suggested advancement of the needle at a 15–30 [10] angle from the frontal plane in pediatric patients. The present case strongly suggests that the needle should not be inserted at, or immediately below, the inguinal ligament, and it should not be advanced too far at a lower angle so as to avoid puncture of the incompressible retroperitoneal vessels and subsequent uncontrollable hemorrhage.

We present a case of acute onset of massive retroperitoneal hemorrhage following inadvertent arterial puncture during femoral vein cannulation attempts. Imaging observation of the retroperitoneal region strongly suggested that the external iliac artery was inadvertently punctured during the cannulation attempts, causing massive retroperitoneal hemorrhage. This theory was further supported by the preoperative radiologic examinations. In our case, the site for insertion of the needle was too proximal and the angle for advancement of the needle too low, enabling the needle to reach and thus injure the incompressible retroperitoneal vessel. The anatomy of the “right” femoral and external iliac vessels suggests a higher risk of “arterial” puncture with the approach used in our case; the artery comes close to, overrides, and finally goes across the vein above the inguinal ligament, allowing a greater risk of arterial puncture when the needle is inserted at a proximal site, directed at a low angle, and advanced too far medially and parallel to the arterial pulsation.

## Author Contributions

**Sadegh Abaei Hasani:** conceptualization, investigation, methodology, project administration, supervision, writing – original draft, writing – review and editing. **Samin Shokravi:** conceptualization, data curation, investigation, project administration, visualization, writing – original draft, writing – review and editing. **Payam Fattahi:** writing – original draft, writing – review and editing. **Niki Tadayon:** investigation, supervision.

## Funding

The authors have nothing to report.

## Conflicts of Interest

The authors declare no conflicts of interest.

## Data Availability

The data that support the findings of this study are available on request from the corresponding author. The data are not publicly available due to privacy or ethical restrictions.
